# Prevalence and Associated Risk Factors of Dry Eye Disease Among Children and Adults in Saudi Arabia: A Cross-Sectional Study

**DOI:** 10.7759/cureus.40170

**Published:** 2023-06-09

**Authors:** Dana A Alrabghi, Rahaf L Abudungor, Yasmeen S Alsulaiman, Albaraa Najjar, Ahmed M Al-Manjoumi

**Affiliations:** 1 Faculty of Medicine, Fakeeh College for Medical Sciences, Jeddah, SAU; 2 Faculty of Medicine, King Abdulaziz University, Rabigh, SAU

**Keywords:** cross sectional, saudi arabia, pediatrics, prevelance, risk factors, dry eye disease

## Abstract

Background: Dry eye disease (DED) is characterized by loss of homeostasis of the tear film or failure to produce sufficient amounts of tears to moisturize the eyes. The condition has been associated with several preventable risk factors.

Objective: The objective of this study is to calculate the prevalence of dry eye and determine the associated risk factors among adults and children in Saudi Arabia.

Methods: This is a cross-sectional study targeting all Saudi populations, including all the regions of Saudi Arabia. The Ocular Surface Disease Index (OSDI) and the five-item Dry Eye Questionnaire (DEQ-5) were used for data collection. Data were collected using an online form that was distributed through social media.

Results: A total of 541 responses were analyzed. The OSDI scores showed that females represented 70.9%, and the age group of 20-40 years represented 59.7%. The prevalence of DED, including all severity levels, was 74.9%. The distribution across levels was as follows: mild cases at 26.2%, moderate cases at 18.2%, and severe at 30.4%. On the other hand, DEQ-5 has shown a prevalence of 37% among the pediatric age group. Several risk factors have been significantly associated with adults' dry eye, including low humidity (P-value=0.002), reading, driving, or watching electronic screens for extended durations (P-value=0.019), autoimmune diseases (P-value=0.033), and undergoing eye procedures (P-value-0.013).

Conclusion: The current study reports a high prevalence rate of dry eyes among the Saudi population. Reading, driving, and using electronic screens for an extended period were found to be associated with the severity of DED. Prospective studies should focus on the epidemiology of the disease, which will provide evidence for better preventive and therapeutic measures.

## Introduction

Dry eye disease (DED) is a common chronic multifactorial condition of the ocular surface characterized by loss of homeostasis of the tear film or failure to produce sufficient amounts of tears to moisturize the eyes [1]. It is a condition that affects the ocular surface, causing pain, visual disruption, tear film instability, and the possibility of ocular surface injury. The tear film's osmolarity rises, and the ocular surface becomes inflamed [2]. Tiredness, burning, itching, photophobia, inflammation, and visual disturbances are all common symptoms of dry eye [3]. While DED can rarely lead to loss of vision, it can diminish the quality of life when symptoms are present. Risk factors include secondhand smoke exposure and prolonged periods of driving or reading or exposure to electronic screens [4,5]. 

Many autoimmune diseases are associated with an increased risk of DED, including rheumatoid arthritis, sarcoidosis, and Sjogren syndrome [4]. Moreover, dry eye is associated with comorbidities in almost all body systems, including musculoskeletal, gastro-intestinal, psychiatric, functional, dermatological, and atopic disorders, keratoconus, osteoarthritis, connective tissue diseases, atherosclerosis, Graves' disease, autistic disorder, depression, 'burnout,' Crohn's disease, sarcoid, lichen planus, rosacea, liver cirrhosis, sleep apnea, sinusitis, thyroid function, and air pollution [6].

Patients with Parkinson's disease are at higher risk of developing DED due to the decreased number of blinks (one to two per minute) compared to healthy individuals (16-18 blinks per minute). This leads to a diminished distribution of the lipid components of the tear film over the cornea, causing the aqueous component to evaporate faster [7]. Previous epidemiological evidence has shown that the overall prevalence of DED is between 5-50% [6]. In Brazil, the prevalence is 12.8% [8]. Factors including geographic, climatic, and demographic aspects have been reported to influence the variation in the frequency of dry eye [9]. A study in Ontario in 2017 estimated that over six million Canadian adults may have DED [10].

A cross-sectional study conducted in 2017 in Dubai, United Arab Emirates (UAE), which included 452 participants, estimated that the prevalence of dry eyes in Dubai was 62.6%, with severely dry eyes being the most prevalent (42%) [11]. Another study conducted in Al-Ahsa, Saudi Arabia, in 2017 concluded the prevalence of dry eye was 32.1% [12]. A population-based survey conducted in Riyadh between 2013 and 2017 concluded that the prevalence of DED was 45.1% of 890 participants [13]. A cross-sectional study conducted in 2019 in Arar, Saudi Arabia, with a total sample of 581 individuals reported that the prevalence of DED was 36.5% [14].

Considering the effect of DED on the quality of life (QOL), the condition may also affect patients' daily routine, like interfering with plans due to the weather and making reading uncomfortable. Numerating diseased patients and identifying risk factors will help patients prevent dry eye situations and seek healthcare services early. To the best of our knowledge, no recent studies assessed the prevalence and risk factors of dry eye among adults and children in Saudi Arabia, especially in the Western region. This study aimed to calculate the prevalence of dry eye and determine the associated risk factors among adults and pediatric patients in Saudi Arabia.

## Materials and methods

A descriptive cross-sectional study was carried out among the general population of Saudi Arabia from August to September, 2021. The data was collected using an online tool sent to the participants through social media. All Saudi citizens of both genders from all regions were included. There were no exclusion criteria. The sample size was calculated using an online calculator for cross-sectional studies (Raosoft, Inc., Seattle, Washington, United States) [15]. The equation was built using a 5% margin of error, 95% confidence level, and 50% response rate. The calculated sample size was 382. However, since the questionnaire was distributed through social media, the total response was 541. Therefore, we used a larger sample size.

Two validated questionnaires were used to assess dry eye disease prevalence and risk factors. Ocular Surface Disease Index (OSDI) for adults and five-item Dry Eye Questionnaire (DEQ-5) for children. OSDI is composed of 12 calculable components giving a total score reflecting the severity of DED; normal (0-12 points), mild (13-22 points), moderate (23-32 points), and above 33 severe. It is divided into three categories, including sensitivity, eye limitation, and feeling uncomfortable. DEQ-5 is composed of five components, mild (8.6 ± 3.1), moderate (11.4 ± 3.3), and severe (14.9 ± 2.3), and divided into three categories, including eye discomfort, eye dryness, and watery eyes.

The data collection tool was distributed via social media platforms such as Twitter (Twitter, Inc., San Francisco, California, United States) and WhatsApp groups (Meta Platforms, Inc., Menlo Park, California, United States), during August 2021. 

The analysis was carried out using IBM SPSS Statistics for Windows, Version 27.0 (Released 2020; IBM Corp., Armonk, New York, United States). Numerical variables showed skewness, for which they were summarized using median and interquartile range (IQR). Proportions were used to summarize categorical variables. The items of OSDI were summed, and the percentage was calculated for all the participants. The computed OSDI scores are considered the outcome variable for gender and various risk factors included in the data collection tool. A non-parametric statistical test (Mann-Whitney U test) was used to find the statistical significance. The significance level was set at p=0.05.

The Institutional Review Board of Doctor Soliman Fakeeh Hospital, Jeddah, Saudi Arabia, approved this study (approval number: 294/IRB/2022). All participants were informed about the aim of the study, which was included on the cover page. The data were handled while confidential and used for research purposes only. 

## Results

A total of 541 responses were analyzed. The age ranged from one year to 72 years old with a median of 32 and IQR of 23-46. Females represented 70.9%, and the majority (85.3%) were from the western region of Saudi Arabia. The sociodemographic characteristics are shown in Table 1.

**Table 1:** Sociodemographic characteristics of the participants

Variable		N	%
Age	<20	58	10.7%
	20-40	323	59.7%
	41-60	124	23%
	>60	36	6.6%
Gender	Male	157	29%
	Female	384	70.9%
Region	West	462	85.3%
	South	14	2.5%
	Middle	37	6.8%
	North	9	1.6%
	East	19	3.5%

OSDI

The OSDI score contained 12 questions assessing the presence and severity of dry eye. Each question was measured using statements describing the frequency of experiencing eye limitation or feeling uncomfortable. Pain, photophobia, and blurry vision were mostly reported in windy conditions, therefore, there were limited functions (reading, driving at night, working with a computer, and watching TV). At the same time, reading and sensitivity to light was the most frequent experience reported during the last week before participation. The 12 items of OSDI are detailed in Table 2. 

**Table 2:** Responses to the OSDI scale OSDI: Ocular Surface Disease Index; ATM: automated teller machine

	None of the time	Some of the time	Half of the time	Most of the time	All of the time
1. Eyes that are sensitive to light?	190 (37.5%)	206 (40.6%)	29 (5.7%)	68 (13.4%)	14 (2.8%)
2. Eyes that feel gritty?	256 (50.5%)	185 (36.5%)	27 (5.3%)	29 (5.7%)	10 (2%)
3. Painful or sore eyes?	194 (38.3%)	204 (40.2%)	42 (8.3%)	51 (10.1%)	16 (3.2%)
4. Blurred vision?	226 (44.6%)	164 (32.3%)	43 (8.5%)	46 (9.1%)	28 (5.5%)
5. Poor vision?	216 (42.6%)	138 (27.2%)	38 (7.5%)	53 (10.5%)	62 (12.2%)
Eye discomfort while:					
6. Reading?	268 (52.9%)	133 (26.2%)	30 (5.9%)	48 (9.5%)	28 (5.5%)
7. Driving at night?	395 (77.9%)	68 (13.4%)	12 (2.4%)	13 (2.6%)	19 (3.7%)
8. Working with a computer or on an ATM?	321 (63.3%)	103 (20.3%)	25 (4.9%)	32 (6.3%)	26 (5.1%)
9. Watching Television?	315 (62.1%)	121 (23.9%)	23 (4.5%)	32 (6.3%)	16 (3.2%)
10. In windy conditions?	138 (27.2%)	159 (31.4%)	42 (8.3%)	91 (17.9%)	77 (15.2%)
11. In places or areas with low humidity (very dry)?	233 (46%)	113 (22.3%)	35 (6.9%)	65 (12.8%)	61 (12%)
12. In areas that are air-conditioned?	200 (39.4%)	153 (30.2%)	30 (5.9%)	62 (12.2%)	62 (12.2%)

The outcome variable was categorized into four categories (normal, mild, moderate, and severe). About one-third (30.4%) had OSDI scores indicating severe dry eye during the last week. The categories of dry eye severity are shown in Table 3. 

**Table 3:** The OSDI score groups OSDI: Ocular Surface Disease Index

OSDI score	N	%
Normal	121	25.0%
Mild	127	26.2%
Moderate	88	18.2%
Severe	147	30.4%

Participants were asked to choose from the list of the risk factors that apply to them. The list contained a total of nine potential risk factors (Table 4).

**Table 4:** The distribution of the reported dry eye risk factors

Risk factors	Yes		No	
	N	%	N	%
I live in an area of low humidity	63	12.4%	444	87.6%
I use beta-blockers or allergy medications	37	7.3%	470	92.7%
I use contact lenses	90	17.8%	417	82.2%
I get exposed to second-hand smoking	123	24.3%	384	75.7%
I read, drive, or use a screen for long	369	72.8%	138	27.2%
I have an autoimmune disease	11	2.2%	496	97.8%
I have Parkinson's disease	1	0.2%	506	99.8%
I have mental illness	110	21.7%	397	78.3%
I have undergone an eye procedure	118	23.3%	389	76.7%
None of the above	66	13.0%	441	87.0%

Further analyses investigated the association of each risk factor with the calculated OSDI scores. The mean rank of the OSDI was used to compare the OSDI scores of the groups. Risk factors were assessed for significance; living in low humidity and using beta blockers or allergy medications showed a higher mean rank of OSDI (P-value=0.002). Reading, driving, or using the screen for a long time, and having an autoimmune disease showed significantly higher mean ranks (P-value=0.019, P-value=0.033, respectively). Mental illness and eye procedures were also significantly associated with dry eye scores (P-value<0.001, P-value=0.013, respectively). Not having any risk factors showed a lower mean rank than having one or more risk factors (P-value<0.001). While not statistically significant, those exposed to secondhand smoke had a higher mean rank than those not exposed (P-value=0.105). The details are given in Table 5.

**Table 5:** Mean rank table for associations OSDI: Ocular Surface Disease Index

Risk factors	OSDI mean rank		P-value
	Yes	No	
I live in an area of low humidity	308	246	0.002
I use beta-blockers or allergy medications	326	248	0.002
I use contact lenses	271	250	0.235
I get exposed to second-hand smoking	273	248	0.105
I read, drive or use a screen for a long time	263	229	0.019
I have autoimmune diseases	347	252	0.033
I have Parkinson's disease	273	354	0.907
I have mental illness	299	242	<0.001
I have undergone an eye procedure	283	245	0.013
None of the above	181	265	<0.001

There is no significant correlation between age groups and OSDI scores (P-value<0.05). Furthermore, females showed a significantly lower mean rank of OSDI scores than males (P-value<0.001). 

DEQ-5

For the pediatric age group, a total of 58 patients were included in the analysis. DEQ-5 score was used for pediatric patients aged 15 years old or less. The age ranged from one year to 15, with a median of 8 and IQR of 5-12. Females represented 53.4% of the total pediatric population. The total scores of DEQ-5 ranged from 0 to 22 with a median of 3 and IQR of 0-8. A cut-off above six points was considered an indicator of dry eye. The prevalence of dry eye was 37% among those aged 15 or under. 

Age showed a moderate correlation with the total score of DEQ-5 (correlation coefficient=0.453), which was statistically significant (P-value<0.001) among the pediatric age group. Upon analysis of dry eye association with gender among the pediatric group, females showed a higher prevalence of dry eye (45.2%) compared to 29.6% among males. However, this finding was not statistically significant. The results are given in Table 6. 

**Table 6:** Association of gender with the results of pediatric DEQ-5 DEQ-5: five-item Dry Eye Questionnaire

	DEQ-5		P-value
Gender	Dry eye (%)	Normal (%)	
Male	8 (29.6%)	19 (70.4%)	0.224
Female	14 (45.2%)	17 (54.8%)	

## Discussion

Sociodemographic, behavioral, environmental, and medical factors can play a crucial role in developing DED symptoms, influencing the patient's QOL. This cross-sectional study aimed to estimate the prevalence of DED and highlights the correlated risk factors among children and adults. In the present study, the prevalence of DED was high (74.9%). This high prevalence was in parallel with another recent study which showed a high prevalence of DED in Saudi Arabia (49.5%) [16].

Although most of our study sample were females, they were found to have a significantly lower OSDI score. This was contrary to many studies that showed that females had a higher risk of developing DED. For example, a recent study conducted in Brazil showed that females were found to be more susceptible to severe symptoms of DED than males (16% and 8.5%, respectively) [17]. In addition, another recent study to assess the prevalence of DED in older people showed that DED was more common in older females [18]. Moreover, a recent Nigerian study revealed that female adults were four times more likely to develop DED [19]. From the authors' perspective, as "reading, driving, or long screen time'' is the most common risk factor among our participants, this could be the reason that males in our study suffer from DED more than females, as driving in Saudi has been predominantly by male drivers [20]; this explains the difference in results between our study and studies in other countries.

In terms of the different age groups, surprisingly, the difference was not significantly correlated with higher OSDI scores in our study. This could be due to the fact that different age groups have different risk factors. However, a recent meta-analysis showed that age is a significant risk factor for DED [21]. A study on the pediatric age group showed that DED was diagnosed in 0.4% of children and adolescents [22]. However, our findings showed a much higher prevalence among the same age group (37%), which can be explained by the usage of a visual display device (VDD) for an extended time, particularly for this age group. Indeed, a recently published study showed that extended screen time was significantly associated with dry eye symptoms, affecting ocular surface health and quality of life in young people [23].

One of the factors that can increase the susceptibility to developing DED symptoms is using eye cosmetics [24]. In our study, there was no correlation between the female gender and DED. This could be due to the precautions and awareness done regarding eye cosmetics hygiene.

A significant association was found between mental illness and DED (P-value <0.001). This could be due to the disturbed lifestyle patients with mental illness may have. Our study showed that a huge number of participants have a mental illness, this could be due to the prevalence of mental illness or due to the misconception and misdiagnosis that individuals have towards mental illness. Other risk factors include low-humid weather, using beta blockers or allergy medication, autoimmune diseases, having an eye procedure, and reading, driving, or using the screen for long times. Furthermore, a recently published systematic review of six articles showed that using contact lenses, exposure to computer screens, thyroid diseases, hypertension, and taking antidepressants and antihistamines were the most crucial and potent risk factors for DED [25]. In addition, a recent meta-analysis of 48 articles also reported various risk factors, including medical conditions such as glaucoma, cataract, hypertension, diabetes, cardiovascular, thyroid, tumors, systemic diseases, VDDs, and contact lens use [26].

Using contact lenses was not significantly associated with a high OSDI score in our study. This could be because the questionnaire does not specify the details of contact lenses use such as the continuous length of time they are worn, whether removed before sleep or not, and if refreshing eye drops are used before and after. However, a review of the literature revealed its significant association with DED. A recent study showed the association between contact lenses and DED symptoms among VDDs users using OSDI scores [27]. The study concluded that DED symptoms were found to increase with using contact lenses. Another study reported that contact lens users are at higher risk of developing DED symptoms [28]. Therefore, it was recommended to consider taking preventive treatment into account for patients using contact lenses. Some of these preventive treatments were studied in a recent study to examine the application of artificial tears and contact lenses, concluding that they significantly reduced DED symptoms [29]. In concordance with our findings regarding the association between secondhand smoke and DED, a systematic review of 22 studies concluded that smoking may not be considered a risk factor for developing DED, and that there is conflicting information regarding smoking's role in DED development [30].

Furthermore, our results show no statistical significance between Parkinson's disease and DED (P-value= 0.907) although a review done in 2017 shows that patients with Parkinson's disease are at higher risk of developing DED [7]. This is because our sample had only one Parkinson’s patient and this is of course not enough to have statistical significance. 

This study used a validated self-reported questionnaire to investigate DED among the general population of Saudi Arabia. However, a few limitations should be pointed out when interpreting the results. First, only temporality and causation can be determined in a cross-section design, which can be considered a limitation in our study., which can be considered a limitation in our study. Moreover, most of our study sample were females and from the western region, which can affect the generalizability of the findings, especially since our questionnaire was distributed online through social media.

## Conclusions

To sum up, the obtained data in this study showed a relatively high prevalence of DED among children and adults in Saudi Arabia. Low humidity, some medications including beta blockers and allergy medications, eye procedures, and reading, driving, and using the screen for long times, in addition to mental and autoimmune diseases, were found to be significant risk factors of DED. Additional prospective research nationally with larger sample sizes is recommended to adequately explain DED's epidemiology. Moreover, an advanced understanding of DED symptoms and risk factors can improve patients' QOL. Implementing periodic clinical screening and developing guidance on the safe use of VDDs may help prevent DED, particularly in the pediatric age group. In addition, community education about risk factors and adopting preventive measures would be preferred in addition to early diagnosis and treatment.
